# Highlights in IBD Epidemiology and Its Natural History in the Paediatric Age

**DOI:** 10.1155/2013/829040

**Published:** 2013-12-24

**Authors:** Marco Gasparetto, Graziella Guariso

**Affiliations:** Unit of Paediatric Gastroenterology, Digestive Endoscopy, Hepatology, and Care of Children with Liver Transplantation, Department of Women's and Children's Health, University Hospital of Padova, Via Giustiniani 3, 35128 Padova, Italy

## Abstract

*Background*. The number of patients of all age brackets diagnosed with Inflammatory Bowel Disease (IBD) has risen dramatically worldwide over the past 50 years. IBD's changing epidemiology suggests that environmental factors play a major role in modifying disease expression. *Aim*. To review studies carried out worldwide analyzing IBD epidemiology. *Methods*. A Medline search indicating as keywords “Inflammatory Bowel Disease,” “epidemiology,” “natural history,” “Crohn's Disease,” “Ulcerative Colitis,” and “IBD Unclassified” was performed. A selection of clinical cohort and systematic review studies that were carried out between 2002 and 2013 was reviewed. Studies referring to an earlier date were also considered whenever the data were relevant to our review. *Results*. The current mean prevalence of IBD in the total population of Western countries is estimated at 1/1,000. The highest prevalence and incidence rates of IBD worldwide are reported from Canada. Just as urbanization and socioeconomic development, the incidence of IBD is rising in China. *Conclusions*. Multicenter national registers and international networks can provide information on IBD epidemiology and lead to hypotheses about its causes and possible management strategies. The rising trend in the disease's incidence in developing nations suggests that its epidemiological evolution is linked to industrialization and modern Westernized lifestyles.

## 1. Introduction

The number of patients of all age brackets diagnosed with Inflammatory Bowel Disease (IBD) has dramatically increased worldwide over the past 50 years. The changing epidemiology of IBD across time and geography suggests that environmental factors are involved in modifying disease expression [1]. Insight into the worldwide epidemiology of this disease is, thus, important to identify geographic patterns and time trends. Recent findings will also make it possible to estimate the global public health burden so that health care resources can be allocated and research can be targeted in specific geographic regions. Information about environmental factors hypothetically affecting the incidence and prevalence of this disease may, finally, lead to prevention interventions when possible.

The aim of this paper is to review the current levels of knowledge concerning IBD epidemiology worldwide and to examine its natural history in paediatric patients.

The first paragraphs of Section 3 of this report will provide an overview of statistics concerning the incidence and prevalence in the general and paediatric worldwide population, considering its two forms, Crohn's Disease (CD) and Ulcerative Colitis (UC) separately. The rest of the section will outline disease trends in specific geographic areas. In Section 4, we will go on to describe the disease's natural history in the paediatric population examining, as before, the two forms separately.

## 2. Methods

A Medline search using the following keywords “Inflammatory Bowel Disease,” “epidemiology,” “natural history,” “Crohn's Disease,” “Ulcerative Colitis,” and “Unclassified IBD” was carried out.

Clinical cohort and systematic review studies performed during the 2002–2013 time frame were reviewed. Studies conducted earlier were also taken into consideration whenever the data outline was considered relevant to this review.

In view of the fact that they are characteristically based on homogeneous diagnostic criteria, practical aspects of phenotypic data collection, and clinical expertise on IBD treatment, population-based studies were given priority in the selection process. Studies based on health administrative databases were also included whenever they clarified epidemiology statistics in particular with reference to paediatric patients [2].

## 3. IBD Epidemiology Worldwide: Data Regarding the General and Paediatric Populations

The etiology of IBDs, which are disorders of chronic inflammation of the gastrointestinal tract marked by episodes of relapse and remission, is not yet fully elucidated. Its two main forms, CD and UC, are diagnosed on the basis of a clinical suspicion and laboratory, radiological, endoscopic, and histological findings [22]. It is not always possible to distinguish between these two forms using the diagnostic tools available, and the term Inflammatory Bowel Disease Unclassified (IBD-U) is currently used to categorise this subgroup of patients with chronic intestinal inflammation. It is important to remember that the misclassification and/or reclassification of the disease can cause diagnostic delays as well as underestimation of IBD's global prevalence and incidence [23, 24].

The reasons behind the rising worldwide trend are not entirely clear [1]. The current mean prevalence of IBD in the general population of Western countries is estimated at 1/1,000 inhabitants [25, 26]. Although there are only few epidemiologic data available regarding developing countries, the disease's incidence and prevalence seem to be increasing over the past 50 years in practically all regions of the world, indicating its emergence as a global disease [27, 28]. The trend appears to have stabilised for the adult population but not for the paediatric one, especially in Central and Southern Europe where it appears to be rising [29, 30].

Both forms of the disease are thought to be linked to a combination of individual genetic susceptibility, environmental triggers, and alterations in the gut microbiome that stimulate an inflammatory response. The changing epidemiology of IBD across time and geographic areas suggests that environmental factors play a major role in modifying disease expression, and its rising incidence in developing nations seems to be linked to industrialization and the Western lifestyle. The most important environmental associations that have been identified until now are cigarette smoking and appendectomy, although neither alone can explain the variation in the incidence of IBD worldwide. Urbanization in some developing countries, diet changes, antibiotics, hygiene status, microbial exposure, and pollution have all been implicated as potential environmental risk factors for IBD, and individual, familial, and community-, regional-, and country-based environmental risk factors could, singularly or together, contribute to its pathogenesis [31].

Estimates of the incidence of paediatric-onset IBD reported around the world vary considerably [24] as do its patterns and distributions in the various age brackets of the paediatric population [32, 33]. Although less prevalent in infants and in very young children, a number of case reports and small population studies have documented disease onset at very early ages. Heyman et al. [34] analyzed the data from a large multicenter registry of children and adolescents affected by IBD in the attempt to answer specific epidemiologic questions. The cohort—58% diagnosed with CD, 29% with UC, and 13% with IBD-U—contained both incident and prevalent cases enrolled over a 2-year period. According to multivariate analysis, the children younger than 8 were 2.5 times more likely to have UC and 3.5 times more likely to have IBD-U than those older than 8. Colonic involvement with or without disease at other sites was more prevalent in the younger patients. Patients with early-onset IBD, in particular very young patients with UC, were more likely to report a family history of IBD. Patients with familial forms (i.e., children whose parents also suffer from IBD) generally develop the disease earlier due to the genetic anticipation phenomenon [35].

Very early IBD onset appears to distinguish a subgroup of patients with unique characteristics [36–38]. Underlying immune deficiencies including glycogenosis, IL-10R deficiency, and other mutations involving cytokines, receptors, or mediators must always be excluded when a child under 5 is being diagnosed. An intestinal IBD-like pattern has been found to be associated in these cases with systemic immunodeficiency within a complex scenario which can even lead to severe life-threatening events (i.e., systemic infections) [39, 40].

Glocker et al. [41] identified mutations in genes encoding the IL10R subunit proteins in patients with early-onset enterocolitis, involving hyperinflammatory immune responses in the intestine. Genetic-linkage analysis and candidate-gene sequencing on samples from two unrelated consanguineous families with children affected by early-onset IBD and functional assays of patients' peripheral blood mononuclear cells were carried out. The investigators identified three distinct homozygous mutations in genes IL10RA and IL10RB, encoding the IL10R1 and IL10R2 proteins in four out of nine patients with early-onset colitis. The mutations abrogate interleukin-10-induced signalling. According to some case studies examining IL-10R deficient patients, hematopoietic stem cell transplantation (HSCT) is generally successful [40–42].

Assessing the incidence of IBD is complicated, especially in the developmental age, as epidemiological investigations tend to ignore cases of IBD-U although they are generally considered an early stage of the disease (in up to 13% of cases and in as much as up to 20% in some Northern European populations) [29, 30, 43]. IBD can also be difficult to recognize, particularly in children when these present atypical symptoms and in third world countries where medical services are lacking.

### 3.1. The Worldwide Incidence of UC and CD in the General and Paediatric Populations

The incidence of UC has been rising in Western countries over the past 50 years, incrementing from 8–14/100,000 to 120–200/100,000 persons over that period [44]. Moreover, research on migrant populations and persons living in developing countries has reported a recent, gradual increase in the incidence of UC. The disease seems to peak between 10 and 18 years and to affect both genders equally [29, 43, 45].

The incidence of CD has also risen significantly over that time period, incrementing from 6–15/100,000 to 50–200/100,000 persons [44]. While initially relatively low, CD incidence has gradually risen to levels that are similar to those of UC [44]. While CD incidence rates seem to have stabilized in most industrialized countries since the 1980s, an increase in the childhood-onset form continues to be noted [3, 4] (Table 1).

CD incidence seems to peak late in adolescence and in young adulthood (up to 25 years of age). A second incidence peak has been noted for both CD and UC at the sixth or seventh decade [29, 43]. Moreover, a secondary peak is reported in several paediatric cohorts in the preschool age group (4-5 yrs of age) [29, 43, 46].

While CD appears to have a predilection for the female sex in the population at large, male predominance has been observed in the paediatric age bracket [29, 43, 45].

### 3.2. The Incidence and Prevalence of UC and CD in the General and Paediatric Populations Specific to Geographic Areas

A recent systematic review by Molodecky et al. [27] examined data from 167 studies concerning Europe (1930–2008), 52 concerning Asia and the Middle East (1950–2008), and 27 concerning North America (1920–2004). The highest annual incidence rate of UC—24.3 per 100,000 person-years—was registered in Europe, followed at 19.2 per 100,000 person-years registered in North America and then at 6.3 per 100,000 person-years noted in Asia and the Middle East. The highest annual incidence of CD was, instead, registered in North America at 20.2 per 100,000 person-years; it was followed at 12.7 per 100,000 person-years by Europe and then at 5 per 100,000 person-years by Asia and the Middle East. The highest reported prevalence values for IBD were found in Europe (for UC, 505 per 100,000 persons; for CD, 322 per 100,000 persons) and North America (for UC, 249 per 100,000 persons; for CD, 319 per 100,000 persons) [27].

### 3.3. Europe

The incidence of IBD appears to be twice as high in Western with respect to Eastern Europe [1], although an increasing incidence has also been noted in the latter area. A prospective, uniformly diagnosed population study by the EpiCom group [5] which analyzed the ECCO-EpiCom inception cohort of IBD patients was interested in investigating a possible East-West gradient in the incidence of IBD in Europe. A cohort of IBD patients attending 31 centers in 14 Western and 8 Eastern European countries providing medical services to a total population of approximately 10.1 million people was studied. Approximately 1500 patients aged 15 years or older were included in the study population. The overall incidence rate ratios in all the Western European centers were 1.9 (95% CI 1.5 to 2.4) for CD and 2.1 (95% CI 1.8 to 2.6) for UC compared with Eastern European centers. The median crude annual incidence rates per 100,000 in 2010 for CD were 6.5 (range 0–10.7) in Western European centers and 3.1 (range 0.4–11.5) in Eastern ones; for UC it was 10.8 (range 2.9–31.5) and 4.1 (range 2.4–10.3), respectively, and for IBD-U it was 1.9 (range 0–39.4) and 0 (range 0–1.2), respectively. The authors concluded that there is indeed an East-West gradient in IBD incidence in Europe [5] (Table 1).

According to the data from the EPIMAD Registry, collected between 1988 and 2007 and referring to a large area of Northern France populated by almost 6 million inhabitants representing 9.3% of the entire French population [47], there was an increase in CD incidence rates which rose from 5.2 cases/100,000 persons in 1988–1990 to 6.7 in 2006-2007 (+29%). The trend seemed to stabilize after a peak of 7.1 was reached in 1997–1999. CD incidence rates in the 10–19-year-old persons increased by 71% rising from 6.5 (1988–1990) to 11.1 (2006-2007). UC incidence rates, instead, decreased during that same period [47].

A sharp increase in paediatric (<16 years) IBD incidence was reported by a Swedish study carried out in a northern Stockholm County between 2002 and 2007 [6]. The increasing incidence in IBD was primarily explained by the rising incidence—registered at 9.2 (95% CI 7.5–11.2)—in CD patients. UC incidence was found to be 2.8 (95% CI 1.9–4.0). A significant increase in the incidence of UC (*P* < 0.05) but not of CD was observed over the study period. The authors concluded that CD continued to be predominant and there was an increase in UC incidence during the study period [6] (Table 1).

A study by Turunen et al. aimed to analyze the incidence and the clinical picture of IBD from 1987 to 2003 in a large paediatric population in Finland [7]. Data were collected from patient discharge and medical records at the country's 2 largest university hospitals. A total of 604 cases of IBD were diagnosed during the 17-year study period: 203 (34%) of these were registered as CD, 317 (52%) as UC, and 83 (14%) as IBD-U. The mean annual incidence rate increased from 3.9/100,000 (95% confidence interval (CI) 2.5–5.8) in 1987 to 7.0/100,000 (CI 5.0–9.4) in 2003 (*P* < 0.001). The majority of cases were patients who are 12 to <15 years old (*n* = 200, 33%); 5.1% were <3 years old, and 14% were <6 years old. IBD-U was most common in young children (29% of all IBD-U patients). Surgical procedures were generally carried out early during disease course [7] (Table 1).

Another Finnish study carried out by Jussila et al. [4] aimed to assess the geographic distribution of IBD in that country and to estimate its nationwide incidence between 2000 and 2007. The register included all new cases of IBD between 2000 and 2007 which received special reimbursement for IBD medication. Overall, 14,214 IBD patients were identified: 10,352 had UC and 3,862 CD. During the study period the mean annual incidence of IBD per 100,000 was 34.0 : 9.2 in CD and 24.8 in UC. The incidence of UC was notably higher in the males (27.8) than in the females (21.9). In CD the incidence increased only slightly and rates did not differ significantly between genders. The incidence of UC increased from 22.1 in 2000-2001 to 27.4 in 2006-2007. The authors concluded that the incidence of IBD is high in Finland with UC being almost three times more frequent with respect to CD. The incidence rate of UC since the year 2000 has increased, while that of CD has remained fairly stable. A North-South gradient was clearly identified for IBD and UC but not for CD [4] (Table 1).

Sawczenko and Sandhu [8] prospectively described the presenting features, disease localisation, and disease growth in newly diagnosed cases of IBD in a multicenter study conducted between June 1998 and June 1999 in the UK and Ireland. A total of 739 new IBD cases of patients younger than 16 were identified. Only one-quarter of the CD cases presented with the “classic triad” of diarrhoea, weight loss, and abdominal pain; nearly half did not report diarrhoea. The median delay from onset of symptoms to diagnosis was 5 months (mean 11 months). Delays were more common in the CD patients and in the younger children. Short stature was noted only in the patients with CD and not in those with UC. One-fifth of the CD cases had small bowel involvement and also significantly reduced stature. Ileocolonic involvement was documented in most of the CD cases, with only a small minority having isolated ileal or isolated colonic disease. Pancolitis was reported in most of the UC cases, with only a very few affected with isolated proctitis [8] (Table 1).

A study by Armitage et al. [9] aimed to analyse the sociodemographic and geographic distribution of paediatric-onset CD in Scotland. Using a national database, 580 Scottish children (<16 years of age at symptom onset) who were diagnosed with IBD between 1981 and 1995 were identified. The incidence of paediatric-onset CD was 2.5 cases per 100,000 population per year (95% CI: 2.0–2.5) for the 1981–1995 time period and it was significantly higher in Northern (3.1, 95% CI: 2.6–3.8) with respect to Southern Scotland (2.1, 95% CI: 1.9–2.4, *P* < 0.001). The incidence of paediatric-onset UC did not, instead, show any north/south variations (*P* = 0.677). While children from more affluent areas had a higher relative risk of developing CD, paediatric-onset UC did not seem to be associated with affluence [9] (Table 1).

A study conducted in the Czech Republic [10] between 1990 and 2001 aimed to assess the paediatric population suffering from IBD and to determine the incidence of CD in children younger than 15 during the study period. The diagnostic criteria were met by 470 IBD patients; 201 of these turned 18 during the study period. CD was diagnosed in 223 patients. The incidence of CD in the patients younger than 15 rose from 0.25/100,000 in 1990 to 1.25/100,000 in 2001. Severe growth retardation was recorded in 6.4% of the adolescents with CD at the age of 18. UC was diagnosed in 202 patients. The criteria for IBD-U were met in 9.8% of all the IBD patients [10] (Table 1).

A study was carried out by Italian investigators utilizing the National Paediatric IBD Register which was instituted in 1996. The data analysed by the study were collected from the time the register was instituted until 2003 [11]. According to those data, IBD was more frequent in children between 6 and 12 years (57%), although 20% developed their first symptoms before the age of 6. In 1.8% of cases a diagnosis was made within the first year of life. Diagnosis of IBD-U was more frequent in children between 0 and 5 (more than twice as high with respect to older children) and accounted for 10–15% of all new diagnoses of IBD. Mean values of the time interval between onset of symptoms and IBD diagnosis were 10.1 months for CD, 9 months for IBD-U, and 5.8 months for UC. Extended colitis was the most frequent form in UC and ileo-colic involvement the most frequent in CD. Upper intestinal tract involvement was present in 11% of CD patients. IC locations were similar to those of UC. Bloody diarrhea and abdominal pain were the most frequent symptoms in UC and IC, and abdominal pain and diarrhea in CD. Extraintestinal symptoms were more frequent in CD than in UC (Table 1).

### 3.4. The Americas

Canada has one the highest prevalence and incidence rates of IBD in the world [48]. A comprehensive review analysing the burden of IBD, its direct and indirect medical costs, and humanistic impact in Canada was recently conducted by Rocchi et al. [12]. Approximately 233,000 Canadians were diagnosed with IBD by 2012 (129,000 with CD and 104,000 with UC), corresponding to a prevalence of 0.67%. Approximately 10,200 new cases are diagnosed annually. IBD is diagnosed at all ages, with typical onset occurring in the second or third decades of life. Compared to the general population, the quality of life of IBD patients was low across all health dimensions and medical costs associated to its care were high [12]. The review by Rocchi et al. highlighted the elevated burden of the disease in that country due to its high prevalence and medical costs. The authors concluded that Canada is characterized by lack of awareness of IBD as a chronic disease, late or inappropriate diagnosis, inequitable access to health care services, costly medication, diminished employment prospects, and limited community-based support [12] (Table 1).

Benchimol et al. [2] used a population-based clinical health administrative database of IBD patients younger than 15 years living in Ontario, Canada, to define paediatric-onset IBD and to describe its epidemiology. The most accurate algorithm was validated with chart data regarding children from 12 medical practices. Identification of children with IBD required four physician contacts or two hospitalisations with International Classification of Disease (ICD) codes for IBD within 3 years' time if they underwent colonoscopy and seven contacts or three hospitalisations within 3 years' time in those without colonoscopy. Age- and sex-standardised prevalence per 100,000 population of paediatric IBD increased from 42.1 (in 1994) to 56.3 (in 2005). Incidence per 100,000 increased from 9.5 (in 1994) to 11.4 (in 2005). Statistically significant increases in incidence were noted in 0–4-year-old persons (5.0%/year, *P* = 0.03) and in 5–9-year-old persons (7.6%/year, *P* < 0.0001) but not in 10–14- or 15–17-year-old persons [2] (Table 1).

A recent study by Kappelman et al. was carried out in North Carolina to determine the prevalence of CD and UC in a commercially insured US population and to compare prevalences across sociodemographic and temporal conditions [49]. Data from three consecutive 2-year cross-sectional studies based on claims data collected from approximately 12 million Americans were analysed. In 2009, the prevalences of CD and UC in children were 58/100,000 (95% confidence interval (CI) 55–60) and 34/100,000 (95% CI 32–36), respectively. In adults, the respective prevalences were 241/100,000 (95% CI 238–245) and 263/100,000 (95% CI 260–266). According to data analysis, IBD prevalences had slightly increased over time. Based on census data, estimated 1,171,000 Americans have IBD (565,000 CD and 593,000 UC). The authors concluded that the burden of IBD has been increasing in the US over recent years [49] (Table 1).

A study by Kugathasan et al. [14] aimed to define epidemiologic and clinical characteristics of newly diagnosed paediatric IBD patients in a large population-based model in Winsconsin. All paediatric gastroenterologists providing care for Winsconsin children voluntarily identified all new cases of IBD during a 2-year period. The incidence of IBD in children living in that state was 7.05 per 100,000, and the incidence for CD was 4.56, more than twice that of UC (2.14). The IBD incidence rate was found to be similar in the different ethnic groups as well as in the sparsely as opposed to densely populated counties. The majority (89%) of new IBD diagnoses were nonfamilial [14] (Table 1).

Basu et al. [15] conducted a survey on 148 IBD patients attending a university gastroenterology clinic in Houston, Texas, between June 1999 and November 2003 to analyse and compare the impact of IBD on the quality of life in the various ethnic groups living in the United States. Whites made up 40%, African Americans 37%, Mexican Americans 20%, and Asians 3% of the total IBD population. African Americans and whites predominantly had CD, while Mexican Americans predominantly had UC. There was no difference between African Americans and Mexican Americans when separately compared to whites in terms of intestinal manifestations of CD and UC. African Americans with CD had a significantly higher incidence of IBD-associated arthritis (*P* = 0.004) and ophthalmological manifestations, notably uveitis (*P* = 0.028), compared to whites. White UC patients had significantly higher incidences of joint symptoms (*P* < 0.0001) and osteoporosis (*P* = 0.001) with respect to Mexican American UC patients. Whites had a stronger family history of IBD and colorectal carcinoma compared to the other ethnic groups. The authors concluded that there are significant differences in IBD subtypes and serologic markers in the different racial/ethnic groups living in the United States [15] (Table 1).

Hispanics are the fastest growing minority in the US. A recent retrospective study [50] aimed to compare IBD presentation in Hispanic and non-Hispanic whites (NHWs) and in US-born and foreign-born Hispanics. The fact that differences in IBD presentation were found in NHW, US-born Hispanic, and foreign-born Hispanic groups in this study underlines the importance of environmental factors in the disease's development. A total of 325 adult patients were included, 208 of whom were Hispanics. The foreign-born Hispanics, accounting for 68% of the total, were diagnosed at an older age than the US-born Hispanics and the NHWs (45 versus 25 and 27, resp., *P* < 0.05). The foreign-born Hispanics manifested more UC than the US-born Hispanics or NHWs (59.9% versus 41% and 28.2%, resp., *P* < 0.05). No difference was noted in the prevalence of extraintestinal manifestations between the Hispanics and NHWs. More cases of upper gastrointestinal tract CD were found in the NHWs (12.5% versus 3.9%, *P* < 0.05). The rate of IBD-related surgical procedures was higher in the NHWs than in the Hispanics (22.9 versus 7.3 surgeries/100 person-years, *P* < 0.01). The Hispanic patients had fewer prescriptions for biologics and immunomodulators with respect to the NHWs (22.2% versus 55.6%, *P* < 0.01, and 35.7% versus 53.8%, *P* < 0.01, resp.) [50].

In two recent retrospective multicenter studies conducted in Argentina and Brazil on cohorts of paediatric patients (*N* = 424) diagnosed with IBD between 1988 and 2007, the proportion of UC and CD was 62% and 24%, respectively, in Argentina, and 52% and 48%, respectively, in Brazil. Age at diagnosis (*P* = 0.076) was similar in the UC (9.1; 4.6–12.0) and CD patients (9.6; 6.9–13.7) [51].

Pancolitis was the most frequent UC localisation both in Argentina (77%) and in Brazil (76%). Ileocolonic CD localisation was predominant both in the Argentine (46%) and Brazilian (37%) population. The most common phenotype of CD (77%) in the Argentine children was inflammatory. Penetrating CD was predominant in the Brazilian children (27%) [51].

### 3.5. Asia

The epidemiologic and clinical characteristics of IBD patients were assessed by Tozun et al. [16] in a large multicenter, countrywide hospital-based study in Turkey. Twelve centers distributed throughout the country registered all new IBD cases diagnosed between 2001 and 2003. The incidence in the referral population during the study period was 4.4/100,000 for UC and 2.2/100,000 for CD. The IBD incidence in Turkey was lower than that in North and West Europe and similar to that in the Middle East. The majority of the IBD cases were diagnosed in young adults (20 to 40 yrs). A characteristic biphasic age distribution was found with one peak between 20 and 30 and another between 50 and 70. Male predominance was noted in both diseases. Family history was positive in 4.4% in the UC and 8.3% in the CD patients. Extraintestinal manifestations were more frequent in the CD patients, with the exception of arthritis which was equally frequent in both the CD and UC patients. The rate of extraintestinal manifestations was lower than that reported in the literature [16] (Table 1).

Abdul-Baki et al. [17] found an age-adjusted prevalence of 53.1 per 100,000 persons for CD and 106.2 per 100,000 persons for UC in a representative Lebanese cohort. The mean annual incidence was 4.1 per 100,000 persons for UC and 1.4 per 100,000 persons for CD (range, 0–6.9/100,000 for both). The prevalence of IBD of this cohort fell in the intermediate range of that reported for white populations in Europe and North America. The mean age at diagnosis for patients with CD and UC was 28.8 ± 11.1 and 32.0 ± 13.4 years, respectively, and a slight female predominance was noted in the patients studied [17] (Table 1).

El Mouzan et al. [18] assessed the medical records of patients in Saudi Arabia younger than 18 who were diagnosed with IBD and monitored over a 10-year period. Fifty consecutive children were diagnosed with IBD between 1993 and 2002 resulting in an estimated incidence of 0.5 cases/100,000/year and prevalence of 5 cases/100,000 persons in the Riyadh region of Saudi Arabia. Most of the children (90%) were Saudi nationals and the female to male ratio was 1 : 0.6. Sixteen percent of the patients in the 5–18-year-old age bracket were younger than 12. Chronic UC was the most common form accounting for 48%, followed by CD and IBD-U in 38% and 16%, respectively. Although the incidence and prevalence of IBD in this report were lower than those in any other population, a comparison with data collected previously indicates that the incidence is increasing [18] (Table 1).

Together with urbanization and socioeconomic development, the incidence of IBD is increasing in China. A prospective, population-based incidence study was conducted by Zeng et al. [13] between July 2011 and June 2012 in Zhongshan, Guangdong, China. All newly diagnosed IBD cases in that region were included. Forty-eight new cases of IBD (17 CD and 31 UC) were identified over the 1-year study period. Age-standardized incidence rates for IBD, UC, and CD were 3.14, 2.05, and 1.09 per 100,000, respectively. Median ages at diagnosis were 38 for UC and 25 for CD. Disease localisation in the CD patients was defined as terminal ileum only (L1) in 24% of the cases, isolated colonic disease (L2) in 6% of the patients, and ileocolonic disease (L3) in 71% of the patients. Twenty-four percent of the CD patients had coexisting upper gastrointestinal (GI) involvement (L4) [30]. Inflammatory (B1), stricturing (B2), and penetrating (B3) behaviour was noted in 65%, 24%, and 12% of the CD patients, respectively. Fifty-nine percent of the CD and 26% of the UC patients had extraintestinal manifestations [13] (Table 1).

Another prospective study conducted between January and December 2010 by Zhao et al. [52] investigated the incidence of IBD in Wuhan, a major city in central China, using population-based methods. New IBD cases were identified in 17 central hospitals providing health care services in central Wuhan. Overall, 131 new cases of IBD were identified during the 1-year period, including 97 cases of UC and 34 cases of CD. The age-adjusted incidence of IBD, UC, and CD was 1.96 per 100,000 (range 1.62–2.30 per 100,000), 1.45 (range 1.16–1.75), and 0.51 (range 0.33–0.68), respectively. Disease was localized in the small bowel only (L1) in 15% of the CD patients, in isolated colonic disease (L2) in 24%, and in the ileo-colon (L3) in 61%. CD phenotypes were inflammatory in 44% of the cases, stricturing in 29%, and penetrating in 24%. In the UC patients 34.5% had proctitis, 44.6% had left-sided colitis, and 19.5% had extensive colitis [52].

According to the two latter studies, the incidence of IBD in China is similar to that in Japan and Hong Kong but lower than that in South Korea and countries of the Western Hemisphere [13, 52].

The Asia-Pacific Crohn's and Colitis Epidemiology Study, a large-scale population-based study, reported that although the incidence of IBD varies throughout Asia, it is still lower than that in the West. IBD can nevertheless be as severe or even more severe in Asia than it is in the West [20]. China has the highest incidence of IBD in Asia (3.44 per 100,000 individuals). The UC/CD ratio was 2.0 in Asia and 0.5 in Australia. Complicated CD (stricturing, penetrating, or perianal disease) was more common in Asia than in Australia (52% versus 24%; *P* = 0.001), and a family history of IBD was less common in Asia (3% versus 17%; *P* < 0.001) (Table 1).

A study conducted by Tsai et al. aimed to delineate the trend in incidence and clinical patterns of childhood IBD in Taiwan [19]. All children admitted to the National Taiwan University Hospital (NTUH) between 1979 and 2000 meeting the criteria for IBD were included. The clinical features and outcomes were analysed retrospectively. Seventeen children (9 females and 8 males, aged 2 months to 18 years) were diagnosed with IBD during the study period. Six (35%) of these had UC and 9 (53%) CD. The cumulative incidence of CD during 1979–1995 was 0.85% and increased to 2.6% during 1996–2000 (*P* < 0.001), while the incidence of UC did not change significantly between the two periods (from 0.85% to 0.99%, *P* = 0.16). The median interval from onset to diagnosis was 7.7 months. Eighty percent of the patients showed moderate to severe disease activity at diagnosis [19].

Pinsk et al. [21] conducted a study which aimed to determine the incidence of IBD and disease subtype in the South Asian paediatric population living in British Columbia, Canada, compared with non-South Asian IBD patients living in the same area. A chart review was carried out with data collected for all patients ≤16 yr of age diagnosed with IBD from January 1985 to June 2005. Seventy-five South Asian patients were diagnosed with IBD, 48% with CD, 33.3% with UC, and 18.7% with IBD-U, as opposed to 71%, 18.8%, and 10.2%, respectively, in the non-South Asian population. The incidence rate for South Asian IBD patients for the 1996–2001 period was 15.19/10 (6.41/10 for CD, 6.70/10 for UC, and 2.08/10 for IBD-U) compared with 5.19/10 for the non-South Asian IBD group (3.69/10, 0.96/10, and 0.54/10, resp.). The South Asian male/female ratio was significantly different from that observed for the rest of the population. According to these data, there was a significantly higher incidence of IBD in the South Asian paediatric population compared with the rest of the BC paediatric population, as well as a different pattern of phenotypic expression, a male predominance, and more extensive colonic disease. Migration and environmental and lifestyle changes seem on the basis of these results to have an effect on the incidence of IBD and disease subtype [21] (Table 1).

## 4. The Natural History of Paediatric IBD

### 4.1. The Natural History of Crohn's Disease

Although the natural history of paediatric CD is characteristically unpredictable [53], the following factors are fundamental in determining disease evolution [54]: disease activity, the rate of recurrence and complications over time, the need for surgery, and its impact on quality of life.

In terms of growth impairment, approximately 50% of adults with CD presenting in developmental age reach a final height that is 10% lower than that in the general population, and in 25% of cases 5% lower than the established target [55].

Representing an important consideration in 28–36% of cases after the first year of treatment, the risk of corticosteroid dependency is similar in adults and children, regardless of concomitant administration of immunosuppressants. While the use of corticosteroids is efficacious in treating an acute phase of IBD, prolonged use should be avoided or limited [55].

Intestinal surgery is required in as high as 80% of CD patients, and a permanent stoma is required in more than 10% [27]. The risk is higher in patients with genotype NOD2-CARD15 and fibrostenosing disease and in those with serum positivity for anti-ASCA antibodies [55].

Postsurgical relapses occur in 20–30% of adult CD patients, and they are endoscopically documented in 43–79% of cases. In children, the global recurrence rate after 5 years is estimated at 50% and varies, depending on disease localization [53].

Patients with CD have higher mortality rates with respect to the general population [44]. Only precocious aggressive therapeutic strategies focusing on treating early recurrent lesions in asymptomatic individuals seem to have a significant impact on progression of this chronic disease [44].

Four hundred and four CD children (<17 years at diagnosis) forming a geographically derived incidence cohort were diagnosed between 1988 and 2002, by Vernier-Massouille et al. [54] and monitored for approximately two years. The most frequent disease location at diagnosis was the terminal ileum-colon (63%). Disease progression was noted in 31% of the children during the followup. Complicated behaviour was observed in 29% of the children at diagnosis and 59% at followup. Kaplan-Meier survival estimates of the cumulative incidence of surgery were 20% at 3 years and 34% at 5 years after diagnosis. Multivariate Cox models showed that both stricturing behaviour at diagnosis and treatment with corticosteroids were associated with an increased risk for surgery, while treatment with azathioprine was associated with a decreased one. Azathioprine was introduced earlier in the course of disease in patients not undergoing surgery than in those patients requiring surgery. The authors concluded that paediatric CD was characterized by frequent recurrences and severe complicated disease behaviour. Immunosuppressive therapy seemed to improve the natural history of the disease and to reduce the need for surgery [54].

### 4.2. The Natural History of Ulcerative Colitis

Both the clinical presentation at the time of diagnosis and the disease course of UC are heterogeneous and variable over time, and colectomy is one of its major risks [56].

The clinical characteristics of UC that influence its course include the extent and the severity of the disease at diagnosis, both of which correlate with a higher rate of colectomy [56]. While colectomy is required in approximately 10%–30% of UC patients [57], mortality in these patients has not been found to be higher with respect to that in the general population [57].

Just as in adult patients, the response to corticosteroid therapy plays an important role in defining the natural history of the disease in the paediatric population [56].

A short-term remission has been found in 75–80% of children and a long-term one in 60% of patients according to a recent report on the efficacy of biological drugs in UC patients [56, 57].

Gower-Rousseau et al. [58] identified 113 UC children (<17 years at diagnosis) in a geographically derived incidence cohort between 1988 and 2002 who were monitored for at least 2 years. At diagnosis, 28% of the patients had proctitis, 35% left-sided colitis, and 37% extensive colitis. Forty-nine percent of the patients showed disease progression. A delay in diagnosis longer than 6 months and a family history of Inflammatory Bowel Disease were associated with an increased risk of disease progression. The cumulative rate of colectomy was 8% at 1 year, 15% at 3 years, and 20% at 5 years following diagnosis. The presence of extraintestinal manifestations at diagnosis was associated with an increased risk of colectomy. In the patients with limited disease at diagnosis, the risk of colectomy was higher in those who experienced disease progression with respect to those who did not [58].

## 5. Discussion

IBDs are an example of pathological entities having a multifactorial etiology. Over the past several decades, advances have been made in understanding the epidemiology of IBD. At the same time, the incidence and prevalence of both CD and UC have been increasing worldwide across pediatric and adult populations and its epidemiology is evolving. It is to be remembered, however, that the rising pattern may also be due to advances in disease detection and recognition and continued environmental alterations and exposures impacting disease onset. At the same time, the disease is emerging in previously low prevalence areas such as the developing world and among emigrant populations moving to industrialized Westernized societies. Genetic mutations that have been detected can explain a number (about 20–30%) of diagnosed cases but the evidence of IBD's rising frequency in industrialized areas of the world also implicates environmental risk factors [1, 29, 30]. The geographical variability in IBD incidence and prevalence may, in turn, reflect a variety of underlying genetic patterns in different populations [31]. As IBD's prevalence and incidence vary among different ethnic groups and the disease's clinical spectrum is still evolving, environmental factors may continue to contribute to its pathogenesis. Industrialization and globalisation seem, in any case, to be associated with the disease's spreading, rising pattern. In view of these considerations, a growing number of gastroenterologists and paediatricians have become interested in the disease's underlying epigenetic mechanisms.

Some gastroenterologists are particularly concerned about exposure to antibiotics in childhood and specific diets such as the “Western” one containing processed, fried, and sugary foods or low in omega-3 fatty acids may be linked to IBD presentation and the common mechanism may be through an alteration of the gut flora [30]. Greater discretion in prescribing antibiotics to children and ensuring that diets are high in omega-3 fatty acids may be appropriate measures especially in high risk families (positive family history for IBD). Future strategies may aim to modulate gut flora either with antibiotics targeting identified noxious microbes or with selective probiotics that can rebalance the gut dysbiosis [30].

Until now study findings have primarily been retrospective and subject to recall bias and for the most part they have been carried out in richer countries that have the means to do so. As IBD is a relatively rare disorder, with complicated interactions between potential inciting agents, very large cohorts with detailed, prospectively collected, environmental exposure data are needed. At the same time large, well-phenotyped cohorts of IBD patients are likewise necessary if we are to study the effects of environmental exposures on disease course.

In view of these considerations and in particular of the disease's ever evolving epidemiology and impact on quality of life, epidemiologic data must be gathered and analyzed following a systematic, rigorous process. As demonstrated in this review, the process of diagnosing and enrolling patients, be they adults or children, is a complex endeavor. Incidence and prevalence figures extrapolated from published studies regarding paediatric patients have, in particular, been found to be extremely heterogeneous. Establishing multicenter national and international registers and networks would be a step forward in the direction of constructing a shared database system utilizing an established, homogeneous criteria.

This review has attempted to examine the quality of epidemiologic data found in the principal studies carried out on the general and paediatric populations and to identify the weaknesses and gaps existing in the information that is presently available. Implementing a shared database system will hopefully lead the way to well-designed studies and to new, important treatment breakthroughs.

## 6. Conclusions

The highest incidence rates of IBD are in Europe and North America, although the overall prevalence of both CD and UC is increasing throughout the world. Assessing the incidence of IBD is, nevertheless, complicated, especially in the developmental age.

Geographical variability in IBD incidence and prevalence might reflect different underlying genetic patterns within different populations. As there are important differences in the prevalence of IBD between different racial and ethnic groups it is possible that the disease's clinical spectrum is still evolving and that environmental factors might be involved in its pathogenesis. Industrialization and globalization seem nevertheless to be associated with a spreading increasing incidence of IBD.

IBD has a significant impact on patients' quality of life and is generally associated with a high financial burden due to elevated medical costs in particular with regard to biologic drugs.

An analysis of data emerging from multicenter national registers and international networking can provide information on IBD epidemiology and lead to hypothesis about its causes and possible management strategies.

## Figures and Tables

**Table 1:** Summary of the main studies on IBD epidemiology that were reviewed for the special issue.

Authors/paper/year [Reference]	Country	Number of IBD patients enrolled	Patients age	Time period of the study	IBD incidence
Benchimol et al., Gut 2009 [2]	Canada (Ontario)	Population-based clinical database	Paediatric (<15 yrs)	17 yrs (1991–2008)	Age- and sex-standardized prevalence 42.1/100,000 (in 1994) 56.3/100,000 (in 2005) incidence 9.5/100,000 (in 1994) 11.4/100,000 (in 2005).

Chouraki et al., Aliment. Pharmacol. Ther. 2011 [3]	Northern France (EPIMAD Registry)	12.084 CD 7428 UC 4656	Paediatric	20 yrs (1988–2007)	CD In 1988–1990: 5.2/100,000 In 2006-2007: 6.7/100,000

Jussila et al., J. Crohn's Colitis 2013 [4]	Finland	Patients with special reimbursement of medications for IBD in the years 1993 (*n* = 10,958) and 2008 (31,703)	Paediatric and adult	14 yrs (1998–2002)	Nationwide point prevalence of IBD 216/100,000 in 1993 and 595/100,000 in 2008.

Burisch et al., Gut 2013. [5]	Europe (31 centers from 14 Western and 8 Eastern European countries)	1515 patients 535 (35%) CD 813 (54%) UC 167 (11%) IBD-U	Adult (≥15 yrs)	—	Overall incidence rate ratios in all Western European centres were 1.9 (95% CI 1.5 to 2.4) for CD and 2.1 (95% CI 1.8 to 2.6) for UC compared with Eastern European centres Median crude annual incidence rates per 100,000 in 2010: CD 6.5 (range 0–10.7) in Western European centres 3.1 (range 0.4–11.5) in Eastern European centres UC 10.8 (range 2.9–31.5) in Western European centres and 4.1 (range 2.4–10.3) in Eastern European centres IBDU 1.9 (range 0–39.4) in Western European centres and 0 (range 0–1.2) in Eastern European centres

Malmborg et al., J. Pediatr. Gastroenterol. Nutr. 2013 [6]	Sweden (Stockholm county)	133	Paediatric (<16 yrs at onset)	6 yrs (2002–2007)	IBD 12.8/100,000 (95% CI 10.8–15.2) CD 9.2 (95% CI 7.5–11.2) UC 2.8 (95% CI 1.9–4.0)

Turunen et al., Inflamm. Bowel Dis. 2006 [7]	Finland	604 CD 203 (34%) UC 317 (52%) IBD-U 83 (14%)	Paediatric (<18 yrs at onset) 12 to <15 yrs: 200 (33%) <3 yrs: 5.1% <6 yrs: 14%	17 yrs (1987–2003)	IBD In 1987: 3.9/100,000 (95% CI 2.5–5.8) In 2003: 7.0/100,000 (CI 5.0–9.4) (*P* < 0.001)

Sawczenko and Sandhu, Arch. Dis Child 2003 [8]	Great Britain and Ireland	739 CD 431 UC 211 IBD-U 86	Paediatric (<16 yrs at onset)	13 mths (June 1998–June 1999)	—
Armitage et al., Gastroenterology 2004. [9]	Scotland	580	Paediatric (<16 yrs at onset)	15 yrs (1981–1995)	CD 2.3 (95% CI: 2.0–2.5) (Northern Scotland 3.1, 95% CI: 2.6–3.8; Southern Scotland 2.1 95% CI: 1.9–2.4, *P* < 0.001)

Pozler et al., J. Pediatr. Gastroenterol. Nutr. 2006 [10]	Czech Republic	470 CD 223	Paediatric (<15 yrs at onset)	12 yrs (1990–2001)	CD In 1990: 0.25/100,000, in 2001: 1.25/100,000

Castro et al., Inflamm Bowel Dis 2008. [11]	Italy	1576 UC 810 CD 635 IBD-U 131	Paediatric (<18 yrs)	8 yrs (1996–2003)	IBD incidence: 0.39/100,000 (1996)–0.89/100,000 (2003)

Rocchi et al., Can. J. Gastroenterol. 2012 [12]	Canada	233,000 CD 129,000 UC 104,000 (5900 children with IBD)	Paediatric and adult (<18 yrs at onset)	12 yrs (1998–2009)	IBD Prevalence 0.67% Incidence: 10,200 cases/yr

Zeng et al., J. Gastroenterol. Hepatol. 2013 [13]	China (Guangdong province)	48 CD 17 UC 31	Paediatric and adult	1 yr (2011-2012)	IBD 3.14/100,000 CD 1.09/100,000 UC 2.05/100,000

Kugathasan et al., J. Paediatr. 2003 [14]	Winsconsin, US	—	Paediatric	2 yrs	IBD 7.05/100,000 CD 4.56/100,000, UC 2.14/100,000

Basu et al., Am J. Gastroenterol. 2005 [15]	Houston, Texas, US	148 Whites 40%, African Americans 37%, Mexican Americans 20%, Asians 3%	Paediatric and adult	4 yrs (June 1999–November 2003)	—

Tozun et al., J. Clin. Gastroenterol 2009. [16]	Turkey	877 CD 216 UC 661	Paediatric and adult	3 yrs (2001–2003)	CD 2.2/100,000 UC 4.4/100,000

Abdul-Baki et al. Inflamm Bowel Dis 2007 [17]	Lebanon	251 UC 142 CD 100 IBD-U 9	Paediatric and adult	5 yrs (2000–2004)	CD 1.4/100,000 UC 4.1/100,000 (range, 0–6.9/100,000 for both)

El Mouzan et al., J. Trop. Pediatr. 2006 [18]	Saudi Arabia (Riyadh region)	50 CD 38% UC 48% IBD-U 16%	Paediatric (<18 yrs at onset) <12 yrs: 16%	10 yrs (1993–2002)	0.5 cases/100,000

Tsai et al., J. Formos. Med. Assoc. 2004 [19]	Taiwan	17 CD 9 (53%) UC 6 (35%) IBD-U 2 (12%)	Paediatric (<18 yrs at onset)	22 yrs (1979–2000)	CD From 1979 to 1995: 0.85% From 1996 to 2000: 2.6% (*P* < 0.001), UC From 1979 to 1995: 0.85% From 1996 to 2000: 0.99% (*P* = 0.16)

Ng et al., Gastroenterology 2013 [20]	Asia-Pacific (8 countries between Asia and Australia)	419 CD 166 UC 232 IBD-U 21	Paediatric and adult	1 yr (2011-2012)	Asia IBD 1.37/100,000 (95% CI: 1.25–1.51) CD 0.54 UC 0.76
					IBD-U 0.07 China 3.44 per 100,000 individuals Australia IBD 23.67/100,000 (95% CI: 18.46–29.85) CD 14 UC 7.33 IBD-U 2.33

Pinsk et al., Am. J. Gastroenterol. 2007. [21]	South Asian population in British Columbia, Canada	75 CD 48% UC 33.3% IBD-U 18.7%	Paediatric (<16 yrs)	21 yrs (1985–2005)	In 1996–2001: IBD 15.19/100,000 CD 6.41/100,000, UC 6.70/100,000 IBD-U 2.08/100,000

IBD: Inflammatory Bowel Disease, CD: Crohn's Disease, UC: Ulcerative Colitis, IBD-U: Unclassified IBD, yrs: years, CI: confidence interval.
